# Combined interleukin-1 blockade for central nervous system inflammation in children with severe cryopyrin-associated periodic syndromes

**DOI:** 10.1016/j.ero.2026.100245

**Published:** 2026-10-03

**Authors:** Özlem Satirer, Anton Hospach, Nadiya Parczyk, Susanne M. Benseler, Jasmin B. Kuemmerle-Deschner

**Affiliations:** Paediatric Rheumatology, Department of Paediatrics and Autoinflammation Reference Centre Tübingen (arcT), University Children’s Hospital Tübingen, Germany; European Reference Network for Rare Immunodeficiency, Autoinflammatory and Autoimmune Diseases (ERN-RITA), Utrecht, The Netherlands; Department of Paediatrics, Klinikum Stuttgart, Olgahospital, Stuttgart, Germany; Department of Pediatric Radiology, Klinikum Stuttgart, Olgahospital, Stuttgart, Germany; European Reference Network for Rare Immunodeficiency, Autoinflammatory and Autoimmune Diseases (ERN-RITA), Utrecht, The Netherlands; Paediatrics, Cumming School of Medicine,University of Calgary, Alberta, Canada; Children’s Health Ireland, University College Dublin,Trinity College Dublin, Ireland; Paediatric Rheumatology, Department of Paediatrics and Autoinflammation Reference Centre Tübingen (arcT), University Children’s Hospital Tübingen, Germany; European Reference Network for Rare Immunodeficiency, Autoinflammatory and Autoimmune Diseases (ERN-RITA), Utrecht, The Netherlands

Cryopyrin-associated periodic syndromes (CAPS) are rare *NLRP3*-mediated autoinflammatory disorders driven by excessive interleukin-1 (IL-1) signalling [1]. Central nervous system (CNS) inflammation, including aseptic meningitis, papilledema and sensorineural hearing loss, characterises severe CAPS and can cause irreversible damage [2]. Although both anakinra and canakinumab inhibit IL-1, anakinra is often preferred for CNS disease because of superior blood-brain barrier penetration; cerebrospinal fluid (CSF) inflammation may persist under canakinumab [3,4]. In Europe, anakinra and canakinumab are licensed for CAPS [5,6]; rilonacept is US Food and Drug Administration (US FDA)-approved for familial cold autoinflammatory syndrome/Muckle-Wells syndrome (FCAS/MWS), and its prescribing information advises against concomitant administration with another IL-1 blocker [7]. Severe CNS disease may require anakinra escalation up to 8 mg/kg/d, sometimes twice daily, with up to 10 mg/kg/d reported in expert cohorts [5,6,8,9]. High-dose daily anakinra increases injection volume, and prolonged high-dose exposure has recently been associated with drug-derived systemic amyloidosis [10]. Concurrent anakinra and canakinumab is used empirically by some experts, but published evidence is lacking. We reported 3 children in whom low-dose anakinra was added to optimised canakinumab because anakinra escalation was not feasible.

Patients with severe CAPS and active CNS inflammation—CSF pleocytosis, papilledema or abnormal brain magnetic resonance imaging (MRI) scan—persisting despite optimised canakinumab were identified from a prospective cohort [11]. Following ethics approval (No 040/2025BO2), anakinra was added to ongoing canakinumab. Response was assessed using clinical features, Physician Global Assessment (PGA) scale, serum amyloid A (SAA) level, CSF cell counts, brain MRI scan, and ophthalmological and audiological assessments [2,3,8].

Three children (1 girl and 2 boys) with severe CAPS and pathogenic or likely pathogenic *NLRP3* variants were included. Initially, all received anakinra 2 mg/kg/d for a median of 4 months. None was refractory: treatment was switched to canakinumab because of severe injection-site reactions and intolerance of daily injections, not lack of efficacy. CNS and systemic disease activity subsequently emerged or recurred. Canakinumab was optimised by interval shortening and/or dose escalation, but CNS inflammation remained inadequately controlled (Table). Anakinra escalation to 3 to 8 mg/kg/d was considered but not pursued because greater injection volumes were expected to aggravate the reactions that had limited adherence; recently described amyloidosis after prolonged high-dose anakinra was an additional safety consideration [10]. Low-dose anakinra was therefore reintroduced with optimised canakinumab: 2 mg/kg/d in patients 1 and 3 and 2 mg/kg every other day in patient 2, with canakinumab 4 to 6 mg/kg every 4 to 8 weeks (Table). PGA scale score decreased to remission-level activity, SAA level fell markedly, and CSF cell pleocytosis improved in all patients, normalising in 2 and falling to 4 cells/µL in one (Fig, A,B). Inner-ear contrast enhancement resolved in patient 3 (Fig, C). At last follow-up, all were in clinical remission, and no adverse events were reported.

**Table:** Demographic characteristics, disease-related features, and treatment response in patients with severe CAPS and CNS inflammation

Variables	Patient 1 (female)	Patient 2 (male)	Patient 3 (male)
**Demographic characteristics of patients**			
Age at symptom onset	2 mos	8 mo	2 y
Age at diagnosis	1 y	9 mo	6 y
Time to diagnosis	10 mos	1 mo	4 y
Family history	Negative	Positive*	Negative

Clinical and genetic characteristics of the patients			
CAPS phenotype	Severe	Severe	Severe
*NLRP3* variant	Y570C	T368M	M664T
Variant classification (ACMG)	Likely pathogenic	Pathogenic	Pathogenic
CNS symptoms			
• Aseptic meningitis	✓	✓	✓
• Developmental delay	–	–	–
• Frontal bossing	✓	–	✓
• Headache/irritability	✓	–	✓
• Hearing loss	–	–	✓
• Papilledema	✓	–	–

Therapy			
Initial IL-1 blockades			
Drug	Anakinra	Anakinra	Anakinra
Dose	2 mg/kg/d	2 mg/kg/d	2 mg/kg/d
Duration	4 mo	2 mo	6 mo
Second IL-1 blockade			
Drug	Canakinumab	Canakinumab	Canakinumab
Maximum dose	6 mg/kg every 4 wk	4 mg/kg every 8 wk	4 mg/kg every 4 wk
Duration	1 y	2 mo	7 y
Combination therapy (anakinra + aanakinumab)			
Age at initiation	2 y	9 mo	13 y
Dose	Anakinra 2 mg/kg/d Canakinumab 6 mg/kg every 4 wk	Anakinra 2 mg/kg every 2 ds Canakinumab 4 mg/kg every 8 wk	Anakinra 2 mg/kg/d Canakinumab 4 mg/kg every 4 wk
Duration	14 mo	7 mo	18 mo
Age at last evaluation	5 y	2 y	16 y
Adverse events	None	None	None

ACMG, American College of Medical Genetics and Genomics; CAPS, cryopyrin-associated periodic syndrome; CNS, central nervous system; IL-1, interleukin-1.

⁎ Positive family history indicates that the patient’s mother carries the *T368M* mutation and has hearing loss.

**Figure:**
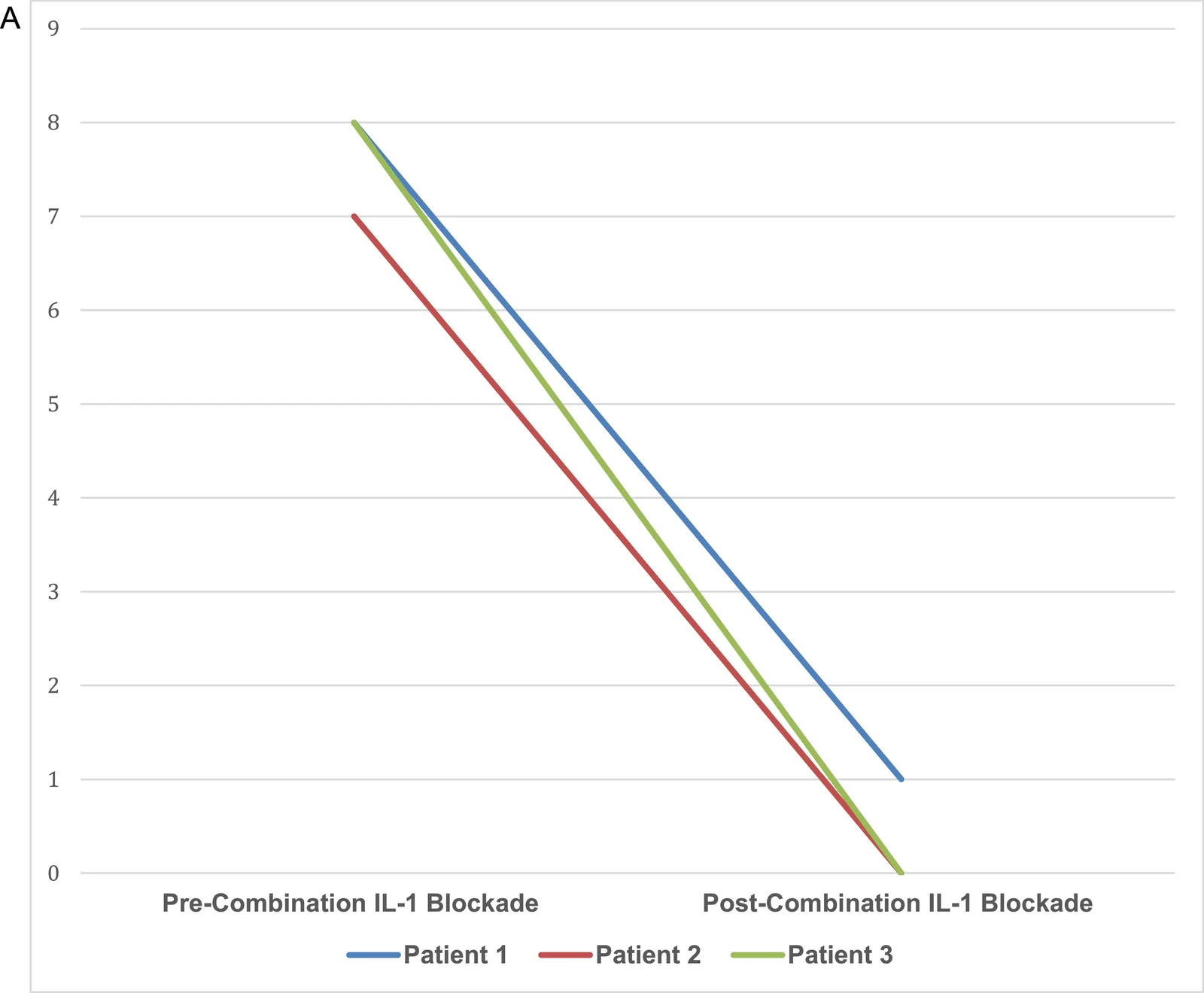

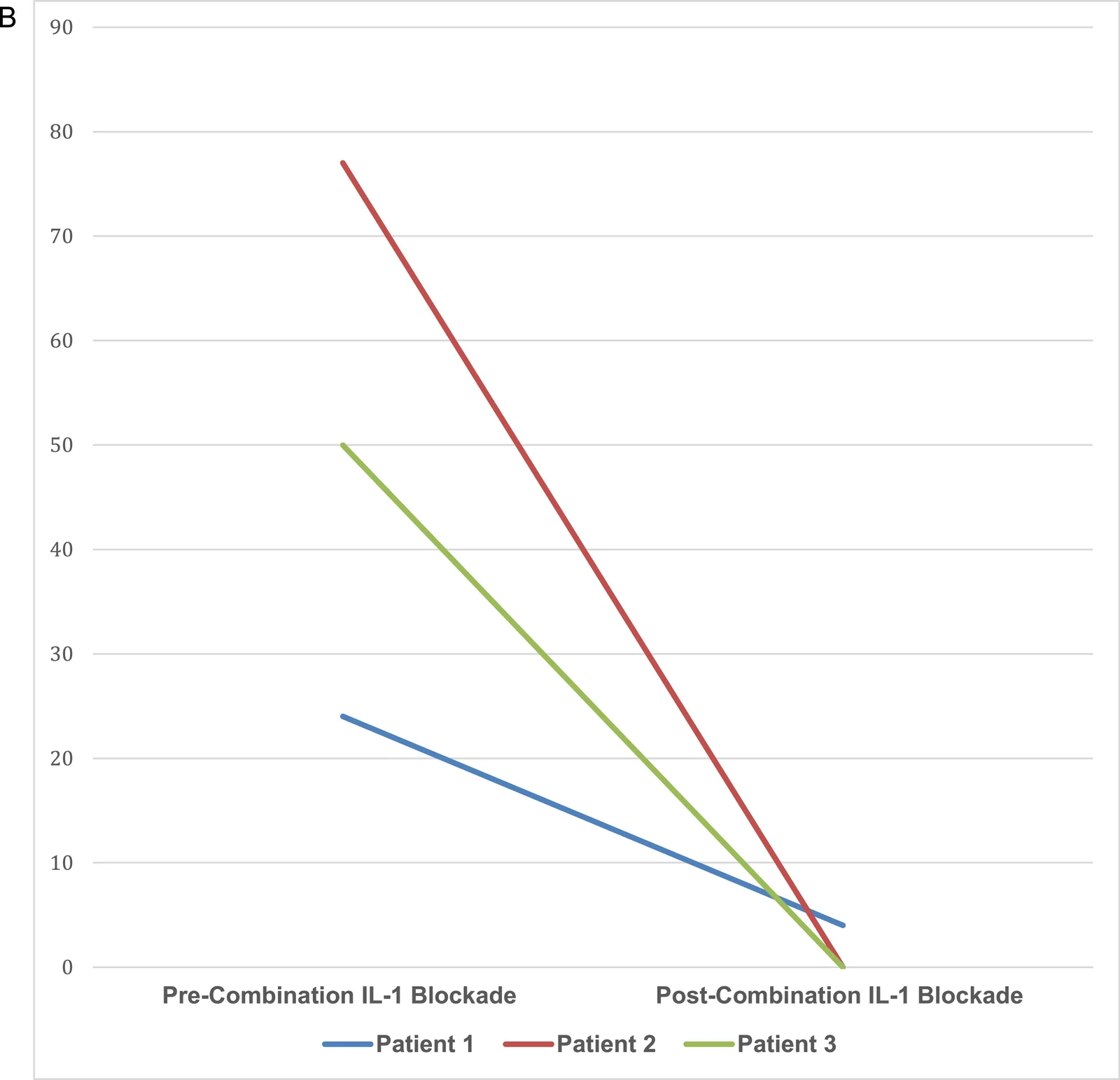

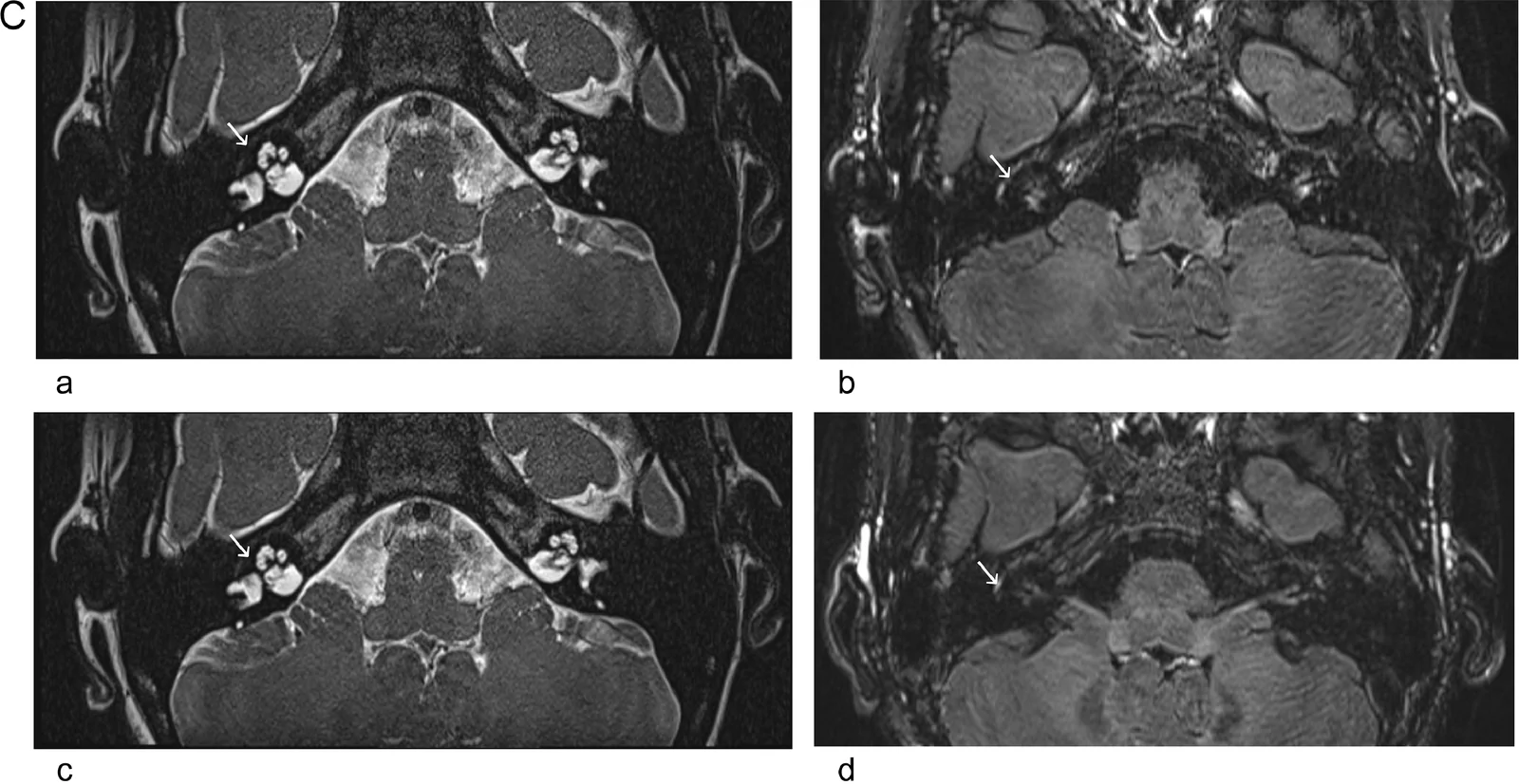
Clinical, laboratory, and imaging findings before and after initiation of combination interleukin-1 (IL-1) blockade. (A) Physician Global Assessment (PGA) scale scores before and after initiation of combination IL-1 blockade. PGA scale score was assessed on a 10 cm visual analogue scale scores (0-10), with scores > 4 indicating high disease activity and ≤2 corresponding to remission-level activity. Prior to combination therapy, all 3 patients had high disease activity (PGA scale score, 7-8) despite ongoing canakinumab monotherapy with interval shortening. Following initiation of combination IL-1 blockade, PGA scale score decreased to 1 in patient 1 and to 0 in patients 2 and 3, reflecting a transition from high disease activity to clinical remission in all patients. (B) Cerebrospinal fluid (CSF) cell counts before and after treatment with combined anakinra and canakinumab in 3 patients with cryopyrin-associated periodic syndromes (CAPS) and central nervous system involvement. All patients showed marked reduction in CSF cell pleocytosis, with complete normalisation in 2 patients (77→0 cells/µL; 50→0 cells/µL) and near-normalisation in one patient (24→4 cells/µL), indicating effective control of neuroinflammation under intensified IL-1 inhibition. (C) Resolution of inner-ear inflammation after combination therapy (Patient 3). (a,b) Baseline magnetic resonance imaging (MRI) scan (September 1, 2023) shows normal T2 signal of the right cochlea on axial short tau inversion recovery (axial STIR). (a) and pathological contrast enhancement in the scala tympani of the basal turn on the right on postcontrast 3-dimensional infrared (3D-IR) images (b), consistent with active inner-ear inflammation during canakinumab monotherapy. (c,d) Follow-up MRI scan (March 2, 2026) after 1.5 years of canakinumab + anakinra combination therapy demonstrates resolution of inflammatory findings, with normal T2 signal on STIR (c) and no contrast enhancement on post contrast images (d).

Adding low-dose anakinra to optimised canakinumab controlled CNS inflammation that had persisted under canakinumab alone; to our knowledge, this is the first published description of combined IL-1 blockade in CAPS, although the approach has been used empirically. Our observations do not establish superiority over adequately escalated anakinra monotherapy, which remains the licensed and guideline-supported option for severe CAPS with neurological disease and should be pursued first [5,6,9]. Concurrent anakinra plus canakinumab is off-label and neither established nor guideline-recommended; here it was an individualised rescue strategy because anakinra escalation was not feasible. Drug-derived anakinra amyloidosis has been described after prolonged high-dose exposure [10], but whether lower cumulative exposure reduces this risk is unknown. Injection-site surveillance, site rotation, and monitoring for proteinuria remain essential [5,6,10]. The long-term safety of dual IL-1 blockade, including potentially additive immunosuppression, is unknown; 3 children followed for 7 to 18 months cannot establish safety. Pooling and sharing of concurrent clinical observations and prospective registry-based studies comparing combination therapy with optimised anakinra monotherapy are needed.

## Contributors

ÖS collected and analysed the data and drafted the manuscript. Anton Hospach provided clinical input and reviewed the manuscript. SMBenseler and JBK supervised the study, interpreted the findings, and critically revised the manuscript. All authors approved the final manuscript.

## Competing interests

JKD has received research grants and speaker’s fees from Novartis and SOBI. The other authors declare no competing interests.
